# Editorial: Early Life Stress-Induced Epigenetic Changes Involved in Mental Disorders

**DOI:** 10.3389/fgene.2021.684844

**Published:** 2021-07-15

**Authors:** Fushun Wang, Fang Pan, Yiyuan Tang, Jason H. Huang

**Affiliations:** ^1^Institute of Brain and Psychological Sciences, Sichuan Normal University, Chengdu, China; ^2^Department of Medical Psychology, Shandong University Medical School, Jinan, China; ^3^Department of Psychological Sciences, Texas Technological University, Lubbock, TX, United States; ^4^Department of Neurosurgery, Baylor Scott & White Health, Temple, TX, United States; ^5^Department of Surgery, Texas A&M University College of Medicine, Temple, TX, United States

**Keywords:** early life stress, mental disorder, epigenetics, emotion, monoamine

Early-life stress is known to be a leading cause of mental illness, and it is a form of chronic social stress that has long-lasting consequences on mental health. However, how exposure to stress during a critical period of development may produce severe long-term neurological changes is still not clear. Epigenetic processes may be one way by which early-life stress becomes biologically embedded, altering how children respond physiologically and behaviorally to stress. The application of epigenetic principles to developmental science is an area of research that has received a great deal of attention in recent years. Some of the most important evidence for epigenetic mediators of early life stems from the work of Brown et al. (2008) who found that early life stress facilitates changes in the cellular methylome at the glucocorticoid receptor gene (Nr3c1) and that these methylations differences persist until adulthood (Weaver et al., 2006). Weaver et al. (2006) provided a mechanistic basis for understanding the down-regulation of the corresponding Nr3c1 mRNA and protein and the attenuated response to subsequent stressors (Brown et al., 2008). Moreover, they have subsequently shown that these differences in DNA methylations are associated with an increased risk for psychiatric disorders later in life. Although new, epigenetics promise to advance the field of child development and research on the effects of early-life stress in several important ways, This is a fast-growing field, and more recent studies have found that several epigenetic mechanisms, including DNA methylation, histone modifications, and miRNA, may play a dynamic physiological role in the adult brain (Kumar and Mohapatra, 2021).

The idea that early life stress leads to mental health problems dates back to Freud, who proposed that psychosis occurs due to traumatic events experienced in early life that are remembered in the subconscious mind. Later on, Bowlby found that early life pressure could cause changes in social relationships, which had been involved in many psychological problems. Bowlby called this relationship attachment, which would affect social relationships in adulthood. Attachment can be divided into guaranteed attachments and non-guaranteed attachments, which could lead to tensions that would cause mental disorders such as phobia, anxiety, or depression (Gu et al., 2016). While the negative effects of early-life stress on children's developmental outcomes are well documented, we know little about how these processes unfold and which children are more susceptible to these exposures. More recently, many investigators have attempted to evaluate the long-term neurological or behavioral changes caused by the stress of early life (Craig et al., 2021). Numerous preclinical and clinical studies show a strong correlation between the HPA axis and the monoaminergic system with aberrant mental health (Wang et al., 2020). Early life stress usually leads to HPA-axis hyper-reactivity in adulthood, leading to monoaminergic dysfunction and, thus, major depression.

Indeed, adversity in early life will have long-term impact on the expressions of many genes, including the HPA axis and monoaminergic transporters or receptors, and monoamine oxidase (Chen et al., 2021). These systems could be affected by epigenetic changes, including methylation of cytosine in genomic DNA or mRNA modification at A, G, and C. Since methylation of cytosine levels influences gene expression and can be long-lasting, altered methylation of cytosine at specific sites or throughout the genome is hypothesized to influence mental and physical outcomes. For example, some early life events induce methylation of the glucocorticoid receptor gene (Nr3c1) and DNA methylation of MAO (monoamine oxidase) (Gu et al., 2016). These epigenetic mechanisms make cell-specific gene expression possible and allow for a genome to be programmed in multiple ways, resulting in a variety of stable gene expression profiles (Kronman et al., 2021). In all, both human and animal studies have shown that monoaminergic neurotransmitter and HPA axis function may be altered by abnormal epigenetic modifications, and these changes will affect the expression of certain proteins under specific stress conditions, and cause affective disorders (Chen et al., 2021). In this special issue, we are pleased to have received 18 total submissions with 10 of them accepted for publication after rigorous peer review. These papers focus on early life traumatic events, which are mediated by epigenetic alternations in affective disorders.

In the paper titled “Early Life Stress- and drug-induced epigenetic modifications of histones in the Ventral Tegmental Area,” Shepard and Nugent provide a focused review on differential histone modifications within the ventral tegmental area (VTA), as well as their relevance to disease-based phenotypes, specifically focusing on epigenetic dysregulation of histones in the VTA associated with early life stress and drugs of abuse. They suggest that locus- and cell type-specific targeting of individual histone modifications at specific genes within the VTA presents novel therapeutic targets that can result in greater efficacy and better long-term health outcomes in susceptible individuals that are at increased risk for substance use and psychiatric disorders.

Wang et al. investigated the molecular mechanisms mediating the effects of early life stress on the development of psychiatric disorders in later life, including anxiety, depression as well as cognitive impairments. They evaluated the changes of mTOR signaling after maternal separation and chronic restraint stress model and analyzed mRNA levels and proteins expression of PSD95 and synaptophysin in the hippocampus. They also examined the activity of mTOR and S6. Their results indicate that maternally separated mice show decreased levels of both mRNA and protein for PSD95, synaptophsin in the hippocampus,as well as decreased phosphorylated mTOR and phosphorylated S6 in maternally separation mice vs. those non-exposed to maternal separation. They concluded that early maternal separation experience impaired synaptic plasticity and inhibited the mTOR signaling pathway, specifically via S6, and thus decreased synaptic plasticity via the inhibition of the mTOR pathway in the hippocampus, which may underlie vulnerability to mental disorders in adulthood. Their research paper is titled “Early-life stress alters synaptic plasticity and mTOR signaling: correlation with anxiety-like and cognition-related behavior.”

In the review paper by Sogo et al., titled “Genetic and epigenetic consequence of early-life social stress on depression: role of serotonin associated genes,” they examined the evidence for early-life social stress-induced epigenetic changes that modulate juvenile and adult social behavior (depression and anxiety). Their review has a particular emphasis on the interactions between early-life social stress and genetic variation of serotonin associate genes, including the serotonin transporter gene (5-HTT; also known as SLC6A4), which are key molecules involved in depression.

Su et al. studied strand-specific next-generation RNA sequencing, and reported in the research article “Gene transcript alterations in the spinal cord, anterior cingulate cortex, and amygdala in mice following peripheral nerve injury.” They observed the changes in whole transcriptomes in the spinal cord, anterior cingulate cortex, and amygdala following unilateral fourth lumbar spinal nerve ligation. They found that the most significantly enriched biological processes amongst the upregulated mRNAs were involved in the immune system process, apoptotic process, defense response, inflammation response, and sensory perception of pain.

In the research article “Differentially expressed genes in the brain of aging mice with cognitive alteration and depression- and anxiety-like behaviors,” Li, Su et al. examined differentially expressed genes and genes with differentially expressed isoforms in the anterior cingulate cortex, amygdala, and hippocampus throughout the lifespan of mice. The RNA sequencing analysis identified 634 and 1,078 differentially expressed genes in ACC, 453 and 1,015 differentially expressed genes in the amygdala, and 884 and 1,054 differentially expressed genes in hippocampus in the 12- and 24-months old mice, respectively. They found that all of these genes with differentially expressed isoforms were closely related to neuroinflammation. They concluded that these neuroinflammation-related genes are likely new targets in the management of memory/cognitive impairment and emotional disorders during aging.

Li, Fu et al. reviewed both clinical and preclinical studies and found that early life stress-induced epigenetic changes of the HPA axis, monoamine, neuropeptides in DNA methylation, histone modification, and RNA transcriptions associated with early life stress might be mediating depression later in life. Epigenetic alterations may add fuel to the fire in the process of depression by blunting the response to antidepressants in those with depression with a history of early life stress. However, they concluded that more research needed to be done to investigate the direct evidence for early life stress-induced epigenetic changes to contribute to the vulnerability of depression, summarized in their review paper, “Effect of early life stress on the epigenetic profiles in depression.”

In the research paper “Long-term Effect of Post-traumatic Stress in Adolescence on Dendrite Development and H3K9me2/BDNF Expression in Male Rat Hippocampus and Prefrontal Cortex,” Zhao et al. established a model of PTSD in adolescent rats using an inescapable footshock procedure. They tested anxiety-like behaviors, social interaction behavior, and memory function with the open field test, elevated plus maze test, three-chamber sociability test, and Morris water maze test. They also examined neuronal development and H3K9me2/BDNF expression in the hippocampus and prefrontal cortex with Golgi staining, Western blotting, qRT-PCR analysis, and CHIP-qPCR analysis. Their results showed that the footshock procedure induced PTSD-like behaviors in rats, resulted in fewer dendrite branches and shorter dendrite length in CA1 of hippocampus and prefrontal cortex, increased H3K9me2 levels, and decreased BDNF expression in the hippocampus and prefrontal cortex.

Wei et al. suggested, in their research paper “Involvement of oxytocin receptor/Erk/MAPK signaling in the prefrontal cortex in early life stress-induced autism-like behaviors”, that the infant period is a critical stage for the development of brain neuroplasticity. They found that juvenile rats subjected to daily 4-h neonatal maternal separation during postnatal day 1 to 20 exhibited autism-like behavioral deficits without impairments in learning and memory functions. Molecular studies showed that oxytocin receptors (OXTR) in the prefrontal cortex of neonatal maternal separation rats were evidently down-regulated when compared with control pups.

In a review paper, Gu et al. (2016) reviewed recent advances in the understanding of the structure and physiological function of monoamine oxidases (MAO), and they also briefly discussed other factors, including stress-induced changes, early life stress, perinatal depression relationship with other epigenetic changes, such as DNA methylation, and microRNA. The paper confirmed the conclusion that early life stress is a significant cause for major depressive disorder, which is one of the leading causes of human disabilities. The paper is titled “Early Life Stress Induced DNA Methylation of Monoamine Oxidases Leads to Depressive-like Behavior.”

In conclusion, epigenetics studies in both animals and humans have given rise to new mechanisms of how early life stressors induce depression-related behaviors. Epigenetic mechanisms, which mediate the life-long effects of perinatal adversity, are potentially attractive targets for early detection, intervention, and prevention of mental health disorders.

## Author Contributions

All authors listed have made a substantial, direct and intellectual contribution to the work, and approved it for publication.

## Conflict of Interest

The authors declare that the research was conducted in the absence of any commercial or financial relationships that could be construed as a potential conflict of interest.
